# Unbiased Estimation of Mutation Rates under Fluctuating Final Counts

**DOI:** 10.1371/journal.pone.0101434

**Published:** 2014-07-02

**Authors:** Bernard Ycart, Nicolas Veziris

**Affiliations:** 1 Laboratoire Jean Kuntzmann, Univ. Grenoble Alpes, Grenoble, France; 2 Laboratoire d'Excellence “TOUCAN” (Toulouse Cancer), Toulouse, France; 3 Sorbonne Universités, UPMC Univ. Paris 06, CR7, Centre d'Immunologie et des Maladies Infectieuses, CIMI, Team E13 (Bacteriology), Paris, France; 4 INSERM, U1135, Centre d'Immunologie et des Maladies Infectieuses, CIMI, Team E13 (Bacteriology), Paris, France; 5 AP-HP, Hôpital Pitié-Salpêtrière, Centre National de Référence des Mycobactéries et de la Résistance des Mycobactéries aux Antituberculeux, Laboratoire de Bactériologie-Hygiène, Paris, France; 6 Mycobacteria Research Laboratories, Department of Microbiology, Immunology and Pathology, Colorado State University, Fort Collins, Colorado, United States of America; Plymouth University, United Kingdom

## Abstract

Estimation methods for mutation rates (or probabilities) in Luria-Delbrück fluctuation analysis usually assume that the final number of cells remains constant from one culture to another. We show that this leads to systematically underestimate the mutation rate. Two levels of information on final numbers are considered: either the coefficient of variation has been independently estimated, or the final number of cells in each culture is known. In both cases, unbiased estimation methods are proposed. Their statistical properties are assessed both theoretically and through Monte-Carlo simulation. As an application, the data from two well known fluctuation analysis studies on *Mycobacterium tuberculosis* are reexamined.

## Introduction

Since the pioneering work of Luria and Delbrück [1], fluctuation analysis has been the object of many studies: see [2]–[7] for reviews. In the past twenty years, the stress has been put on the estimation of the expected number of mutations, for which reliable methods are now available [8]–[15]. However, as Stewart puts it (p. 1140 of [4]):

The parameter 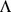 [expected number of mutations] is not, in itself, of biological interest because the experimenter can vary it at will simply by changing the size of the culture vessel or the richness of the medium. What he really wants to know is not 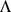, but the mutation rate.

Deriving a mutation rate (i.e. the probability for a mutation to occur upon any given cell division) from an expected number of mutations seems easy: the former is the quotient of the latter by the final number of cells at the end of the experiment. The problem is the definition given to “final number of cells”. The simplest view is expressed by Kendal and Frost (p. 1062 of [2]).

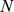
 is obtained by averaging the final number of cells from each parallel culture.

Other authors have developed a more cautious approach, like Foster (p. 198 of [5]).

The validity of the mutation rate calculation requires that 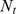 be the same in each culture. Usually, but not always, this can be accomplished by growing cells to saturation. If achieving an uniform 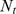 is a problem, the cell number in each culture can be monitored before mutant selection by measuring the optical density or by counting cells microscopically (e.g. using a Petroff-Hausser chamber). Because there is currently no valid method to correct for different 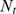
's, deviant cultures must be eliminated from the analysis.

Even under the most careful monitoring, final numbers of cells vary [16]. Yet, final number data are rarely reported in fluctuation analysis experiments, although exceptions exist such as [17], [18]. Theoretical models considering variations in the population size have previously been proposed by Angerer [19] and Komarova *et al.* [20]. Yet, to the best of our knowledge, Foster's assertion that “there is currently no valid method to correct for different 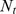
's” remains true to this date. This paper proposes several such methods.

As we shall see, dividing an estimated expected number of mutations by a mean final number of cells, induces a negative bias on mutation rates. Not only the mutation rate, but also the variance of the estimator are underestimated, thus potentially inducing wrong conclusions in statistical testing. Two levels of knowledge on the fluctuations of final numbers are considered. Either the mean and variance of final numbers have been estimated separately, or the final number is known for each culture. In the first case, if 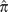 denotes the estimate of the mutation rate assuming constant final numbers, the unbiased estimate 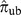 is obtained by:
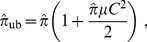
(1)where 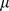 and 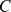 denote the mean and coefficient of variation of the final number of cells. When final numbers are known for all cultures, better results are obtained by the Maximum Likelihood method. The qualities of the proposed estimators have been assessed on a simulation study. The impact on real experiments is discussed, using *Mycobacterium tuberculosis* data published by David [17], and Werngren & Hoffner [21]. Our R [22] implementation of the simulation function and the different estimators is provided in File S1.

## Results

### Simulation experiments

**Figure 1:**
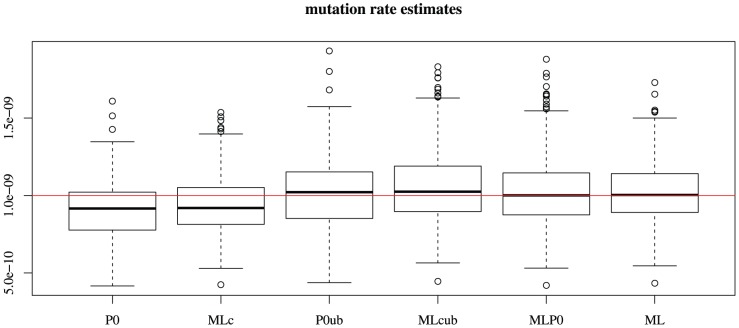
Estimates of a mutation rate on 1000 samples of size 50 of pairs mutant counts – final counts. The horizontal line marks the true value. The first two boxplots correspond the traditional 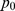
- and ML methods, which estimate the expected number of mutations from the sample of mutant counts, then divide by the final number of cells, supposed as known. On the next two boxplots, the estimates have been multiplied by the unbiasing factor (1). The last two boxplots use the full samples of pairs but no prior knowledge on final numbers. The best results are obtained by the maximum likelihood method (last boxplot). The 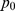-method (label MLP0) performs nearly as well.

Six different estimates of 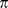 were computed on 1000 simulated samples of 50 couples mutant counts – final numbers. Our choice for the sample size was motivated by two opposite reasons. On the one hand, sample sizes in practice rarely exceed a few tens. On the other hand, confidence interval calculations are all based on asymptotic normality, which requires the sample size to be large enough. A sample size of 50 seemed a reasonable compromise. Boxplots for the estimates are represented on Figure 1. The first boxplot corresponds to the 1000 estimates by the 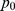-method, assuming the mean final number is known; it is negatively biased as predicted by the theory. The next boxplot represents estimates from the Maximum Likelihood method with known mean final number; it is coherent with the previous one, and similarly biased as expected. On the next two boxplots, the estimates have been multiplied by the unbiasing factor (1). The unbiasing is correct for both methods. For the last two boxplots, each estimate has been computed using the 50 couples with no prior knowledge on the mean and coefficient of variation of final numbers. The best results are obtained by the maximum likelihood method (last boxplot). The 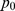-method (label MLP0) performs nearly as well. Since the last two boxplots do not use any prior information, one could have expected their dispersions to be higher than those of the first four. This was not the case, which proves that prior knowledge on the distribution of 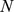 is not a real improvement over measuring final numbers for each culture.

Each estimation method returns a (theoretical) standard deviation, from which confidence intervals can be computed. It is is based on a large sample approximation. The sample size in current fluctuation analysis experiments usually ranges from 20 to 50. Since the estimated standard deviation is of high importance for statistical decision, it was necessary to check whether theoretical standard deviations matched observations. On the same samples, the empirical standard deviation of the 1000 estimates was computed, and compared to the mean value of theoretical standard deviations. For each of the estimators, the theoretical standard deviation was smaller than the observed one; yet, the relative error was smaller than 5%, which validates the theoretical value. For instance, the empirical standard deviation for the maximum likelihood estimate (rightmost boxplot of Figure 1) was 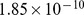, whereas the theoretical value was 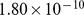.

### Published data sets

In the two references studied here [17], [21], the authors used Luria & Delbrück's method of the mean. Luria & Delbrück [1] themselves had remarked that the method is very sensitive to the size of jackpots and induces important biases; see also Lea & Coulson [23], and Pope *et al.* [6] for a more recent reference.

Table 1 reports mutation rate estimates for the data in Table 1 of David [17]. Since detailed data were not avaible, only the 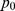-method could be used. The second column contains the author's estimates. The next two columns contain the unbiased 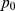-estimate and its 95% confidence interval. Observe that, even though confidence intervals are large due to the small sample sizes, the author's estimates are outside the confidence interval in 5 cases out of 10. The most important discrepancies are due the author's use of a strongly biased estimation method: when large jackpots appear in the mutant counts, as in the Ethambutol cases (last two lines of Table 1), the method of the mean may overestimate 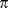 by several orders of magnitude. The main conclusion of [17] was a significant difference in mutation rates, depending on the drug (Isoniazid, Streptomycin, Rifampin, or Ethambutol). Indeed that difference is confirmed by an ANOVA of the estimated mutation rates (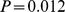).

**Table 1:** Mutation rate estimates from Table 1 of [17].

Determination	Author	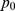 -method	Confidence interval
Isoniazid 1	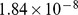	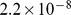	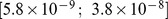
Isoniazid 2	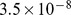	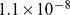	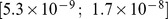
Isoniazid 3	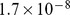	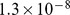	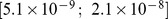
Isoniazid 4	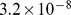	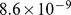	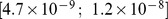
Streptomycin 1	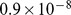	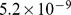	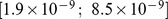
Streptomycin 2	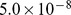	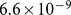	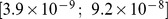
Rifampin 1	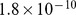	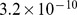	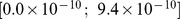
Rifampin 2	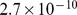	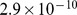	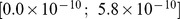
Ethambutol 1	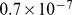	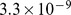	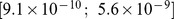
Ethambutol 2	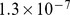	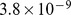	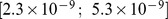

The author's estimates were calculated by Luria and Delbrück's method of the mean. Our estimates were calculated by the 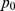-method. The bias correction (1) was applied, with a coefficient of variation 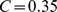 on final numbers. The 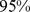 confidence interval is given in the last column.

Table 2 of David [17] contains two paired samples of mutant counts and final numbers. All possible estimates were computed. Values ranged between 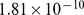 and 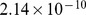. The two values that we consider most reliable, obtained by the maximum likelihood method, were very similar: 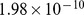 and 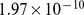. The estimate reported by the author is 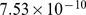. Again, the difference is due to the bias induced by the author's estimation method.

**Table 2:** Mutation rate estimates from Table 1 of [21].

Strain	Authors	ML method	Confidence interval
H37Rv	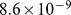	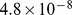	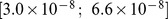
E 865/94	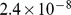	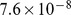	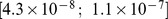
E 729/94	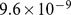	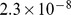	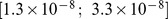
E 740/94	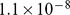	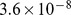	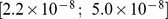
E 1221/94	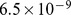	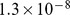	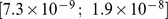
E 1449/94	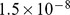	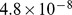	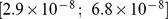
Harlingen	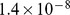	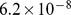	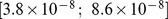
E 26/95	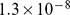	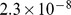	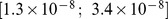
E 80/95	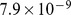	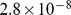	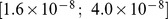
E 55 94	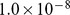	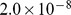	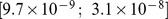
E 26/94	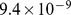	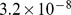	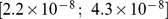
E 3942/94	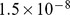	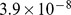	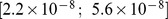
E 47/94	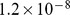	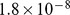	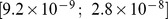

The authors' estimates were calculated by Luria and Delbrück's method of the mean. Our estimates were calculated by the maximum likelihood method under exponential division times. The bias correction (1) was applied, using a coefficient of variation 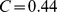 on final numbers. The 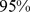 confidence interval is given in the last column.

Table 2 reports mutation rate estimates by the ML method, from data in Table 1 of Werngren & Hoffner [21]. The second column contains the authors' estimates, calculated by Luria & Delbrück method of the mean. The next two columns contain the unbiased ML estimate and its 95% confidence interval. Except for two strains, the authors' estimate is outside the confidence interval. Here, the method of the mean used by the authors has underestimated the mutation rate, because of the very small number of jackpots in the data. The main conclusion of [21] was that no significant difference had been observed between non-Beijing strains (first seven lines) and Beijing strains (last six lines). Actually, the average mutation rate over the first seven lines is 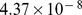, over the last six lines it is 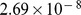. The difference is significant at threshold 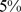 (Welsh Two Sample t-test, 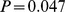).

## Discussion

In any estimation problem, three levels must be distinguished: the reality which is and will remain unknown, the mathematical model which involves more or less realistic hypotheses, and the estimation method. Minimal requirements for an estimator are consistence (outputs should be close to the unknown value of the parameter), and a computable asymptotic variance (to allow statistical inference). Since there is no way to validate all mathematical hypotheses that define the model, another quality is desirable: robustness. Indeed, designing an estimator for a given model and applying it to a different one usually induces a bias: the smaller the bias, the more robust the estimator. For mutation rate estimates, several sources of bias have been identified, such as cell deaths [19], [24]–[26], unknown division time distribution [15], etc. Since there is no way to double check estimates on real data, the usual approach for evaluating an estimation method consists in repeating in silico experiments, i.e. simulate mathematical models for a given value of the parameter, estimate that value repeatedly, and study the distribution of the obtained estimates. A general simulation algorithm described in [15] permits extensive Monte-Carlo experiments.

Usually, only the expected number of mutations is considered as the parameter of interest. Among the many estimation procedures that have been proposed, we have focused on the 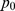-method and the maximum likelihood (ML); they satisfy the basic requirements of statistical inference. As for most other parametric estimation problems, the ML method is the most precise. Provided cell deaths are neglected, the 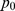-method stands out as the most robust.

All estimation methods are valid only if all observed mutant counts come from the same Luria-Delbrück distribution, i.e. if they have been obtained under a fixed expected number of mutations. However, the parameter of *real* interest which must be considered as fixed, is the mutation rate. For each culture the expected number of mutations is the product of the mutation rate by the final number of cells. Since final numbers vary from one culture to another, so do expected numbers of mutations. As shown here, applying the 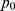
- and ML procedures to the fluctuating final number case as if final numbers were constant, induces a bias. Two solutions have been proposed. In the case where the final numbers of each culture are unknown, but a coefficient of variation is available, an unbiasing factor has been defined, and validated on simulation experiments. The unbiasing factor (1) measures the error induced by neglecting final number fluctuations: the relative error is of order 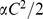 where 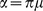 is the expected number of mutations and 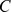 the coefficient of variation of final numbers.

The more favorable case is when final numbers are available. Of course measuring the final number of cells for each culture leads to reducing the volume of the culture in which the mutants are counted, and therefore underestimating mutations. This should be accounted for, by proportionally adjusting the estimates of final numbers. When coupled mutant counts – final numbers have been collected, variants of the 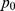
- and ML methods are available. Both yield quite precise estimates. As in the constant final number case, the 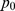-method is more robust, and almost as precise as the ML method. Only the ML method can output relative fitness estimates.

Does the correction for fluctuating final numbers have an impact on the interpretation of the data? We have reexamined the data in two examples chosen from the literature. In both cases, important discrepancies were oberved, that do not only come from neglecting final numbers: they are essentially due to the author's use of Luria-Delbrück's method of the mean, which is very sensitive to jackpots, and can bias the mutation rate estimate by several orders of magnitude. In David's paper, the ethambutol mutation rate had been estimated around 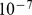 whereas our estimation is of order 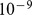. The demonstration is even more striking in Werngren and Hoffner's paper. They compared mutation rate between Beijing and non Beijing *M. tuberculosis* strains and concluded that it was not different and thus could not explain the strong association between Beijing strains and multidrug resistance phenotype. However we re-calcutated the mutation rate and showed that it was significantly higher for Beijing vs. non-Beijing strains. This result is consistent with a recent paper [27] showing that lineage 2 (Beijing) *M. tuberculosis* strains have a higher mutation rate than lineage 4 (non-Beijing) strains. Given the importance of mutation rates on the risk of selection of drug resistant mutants, an accurate evaluation is very important. We hope that our results will help improving precision in the evaluation of mutation rates.

## Conclusion

Dealing with classical estimation methods, Foster [5] was right in recommending that cultures with deviant final numbers be eliminated from fluctuation analysis. Indeed, under varying final numbers those methods underestimate mutation rates, and the relative bias is proportional to the squared coefficient of variation of final numbers. Yet, instead of being discarded as a nuisance, variations in final numbers should be added to the available information to improve estimation: the best mutation rates estimates are obtained when couples mutation count – final number are used.

Two possibilities exist. If mutant counts contain enough zeros (say 10% or more), the 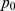-method gives reliable results in virtually null computer time, and is robust both to relative fitness and division time distribution changes. If mutant counts do not contain enough zeros, or if an estimate of relative fitness is sought for, then the joint estimation of the mutation rate and relative fitness should be carried through by the maximum likelihood method.

We are currently working on an optimized implementation of these methods into a forthcoming R [22] package that will be made freely available.

## Methods

Here, 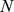 denotes the final number of cells in a Luria-Delbrück fluctuation analysis experiment. Contrarily to the traditional point of view [5], fluctuations on 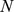 are considered, i.e. 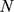 is viewed as a random variable. In the following subsections, different levels of information are assumed on the distribution of 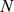: either its Laplace transform is known, or only its expectation and variance are known, or nothing is known, but the final numbers of cells have been measured together with mutant counts for each experiment. Notations for the different parameters are summarized in Table 3.

**Table 3:** Parameters and notations for the mathematical model.

known parameters	
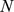	random final number of cells
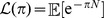	Laplace transform of 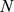
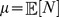	expectation of 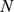
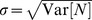	standard-deviation of 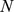
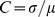	coefficient of variation of 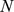
unknown parameters	
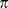	mutation rate
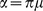	expected number of mutations
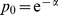	probability of zero mutant
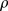	relative fitness of normal cells compared to mutants

Notations for known and unknown parameters: 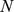 denotes a generic random final number of cells.

As usual, adding a ‘hat’ to the notation of a parameter denotes an estimator of that parameter. We shall consider only strongly consistent, asymptotically Gaussian estimators. If 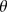 is any parameter, and 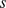 denotes the sample size, then 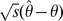 converges to a centered Gaussian distribution as 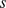 tends to infinity. The variance of that distribution, called asymptotic variance of 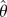, will be denoted by 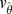.

In the next four subsections, the focus is on the so-called 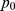-method, introduced by Luria and Delbrück [1] (see also [5], [28]). The problem of jointly estimating the mutation rate 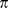 and the relative fitness 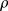 by he maximum likelihood method will be treated after.

### Unbiasing 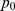-estimates

The final number of cells 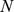 is viewed as a random variable with probability distribution function 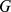 on 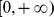. The distribution of 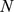 is supposed to be known and its Laplace transform is denoted by 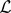.
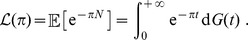



The expectation and variance of 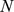 are denoted by 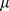 and 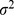 respectively. Let 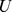 be a random variable, with uniform distribution on 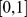, independent from 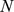. The indicator 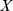 for the mutant count being null is defined as:
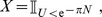
where 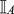 denotes the indicator of event 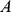 (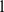 if A is true, 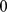 else). Therefore:
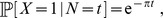
and
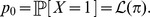



Consider a sample of size 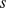, i.e. 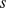 independent copies of 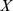: 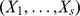. Denote by 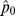 the empirical mean of the 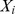
's, i.e. the relative frequency of zeros among mutant counts.
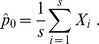



By the central limit theorem, 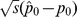 converges in distribution to the centered Gaussian distribution with variance 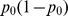, i.e. 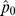 has asymptotic variance 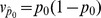.

The 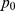-method consists of estimating the mean number of mutations 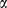 by the negative logarithm of 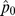, then divide by 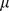 to obtain an estimate of 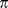.
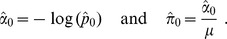



Actually, 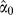 is a consistent estimator of:
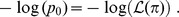



If 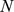 is constant, then 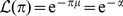, and 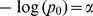: in that case 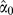 is asymptotically unbiased. If 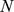 is not constant, because of the convexity of the exponential, and by Jensen's inequality, 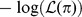 is smaller than 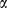, i.e. 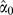 underestimates 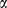, and therefore 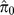 underestimates 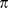.

Denote by 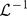 the inverse of 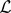 (assumed to be injective). Define a new estimator of 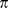 by:
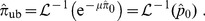
(2)


By construction, 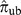 is a strongly consistent estimator of 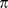, and therefore it is asymptotically unbiased. Its asymptotic variance is obtained by the traditional delta-method (see e.g. [29]): 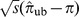 converges in distribution to the univariate centered Gaussian distribution with variance:
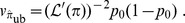



As expected, if 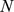 is constant at 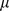, then 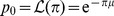, 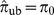, and
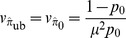



This formula is not new: the asymptotic variance of 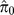 appeared as formula 35, p. 276 of Lea & Coulson [23]; see also [5], [28].

Families of distributions for which explicit expressions of 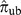 and 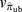 can be obtained are scarce. Two examples are given below.

#### Gamma distributions

They depend on two parameters, usually denoted by 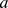 and 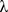. The expectation and variance are:
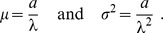



The squared coefficient of variation is the inverse of the shape parameter: 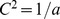. The Laplace transform at 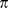 is:
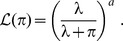



One gets:
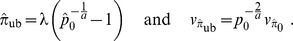



Expressed in terms of 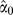, 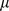 and 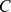:
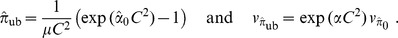



#### Inverse Gaussian distributions

They depend on two parameters, 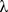 and 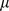. The parameter 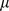 is the expectation, and the variance is 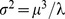. The squared coefficient of variation is 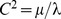. The Laplace transform at 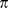 is:
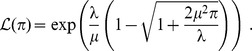



One gets:
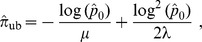
and
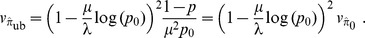



Expressed in terms of 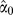 and 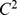, these expressions become:
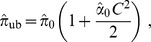
and
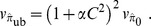



As we shall see in the next subsection, the last two expressions, which are exact for inverse Gaussian distributions, hold as a first order approximation for any distribution.

### First order approximation

If the probability distribution of 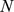 is known, the bias can be exactly corrected by inverting the Laplace transform of 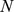. However, this is only a theoretical viewpoint. The best that can be hoped for in practice is an estimate of the expectation of 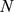 together with its variance. It turns out that whatever the distribution of 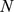, and provided the product of the coefficient of variation by the expected number of mutations remains relatively small, the bias can be corrected. Here, we only assume that the first two moments of 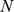, 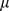 and 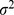 are known, but the full distribution of 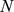, and in particular its Laplace transform, remains unknown. As we have seen, the expectation of 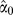 is 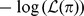. Consider the terms of the series expansion of 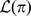 in 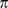 up to order 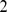 (see e.g. [30]):
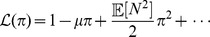



Taking negative logarithm,
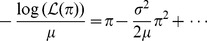



Expressed in terms of 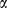 and 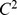, the relative bias is:
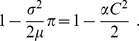



To unbias 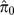, one must divide by the relative bias or (as a first order approximation), multiply by 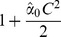. Hence (1):
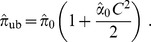



The asymptotic variance, obtained through the delta-method is:
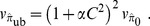
(3)


These expressions are exact for inverse Gaussian distributions, only approximations for any other distribution.

**Figure 2:**
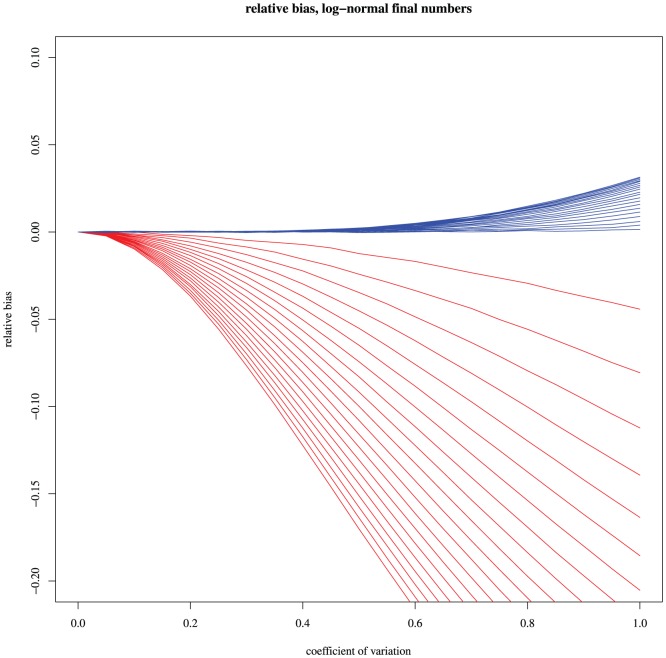
Relative biases on estimates of a mutation rate. Relative biases are plotted as a function of the coefficient of variation 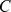. The different curves correspond to 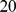 values of 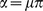 from 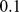 to 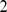. Red curves show biases of the 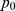-method. For blue curves, the bias has been corrected by the unbiasing factor (1). The correction maintains the bias under acceptable values even for relatively large 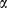 and 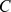.

To assess the validity range of the unbiasing factor, a simulation experiment was conducted. For the same value of 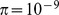, samples of final numbers were simulated with a log-normal distribution with mean 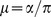 and coefficient of variation 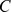. The values of 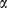 ranged from 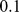 to 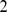, those of 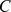 from 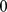 to 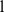. The results are shown on Figure 2. Red curves show the actual relative bias of the 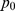-method; for blue curves, the bias has been corrected by the unbiasing factor (1). The correction maintains the bias under acceptable values even for relatively large 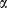 and 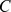.

### The 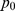-method by maximum likelihood

In this section, nothing is assumed about the distribution of 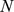. A couple 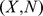 of random variables is considered, where 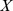 represents the indicator of a null mutant count, and 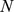 the total number of cells at the end of the experiment. The conditional distribution of 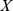 knowing 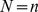, is defined as before:
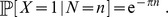



Assume that 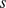 experiments have been repeated independently, yielding 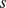 couples 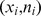, where 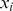 is 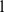 or 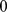 according to whether zero or a positive number of mutants have been counted, and 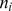 is the final number of cells. The likelihood is the probability of the observation:
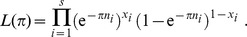



The likelihood depends only on the products 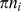. If all 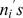 are divided by a given constant, then the maximum likelihood estimator will be multiplied by the same constant. Since the 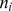
's are very large and 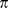 very small, rescaling both can make the calculation numerically more stable.

The log-likelihood and its derivatives are:
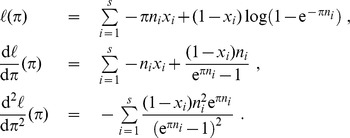



The maximum likelihood estimator 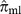 is the solution of 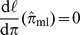, and its asymptotic variance is computed from 
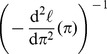
 (see [29]). This is essentially the method used by de la Iglesia et al. [18] in a similar case.

### Bivariate maximum likelihood estimation

In cases where no null mutant counts have been observed, or if an estimate of the relative fitness is desired together with the mutation rate, another procedure must be used. Estimating the two parameters of a classical Luria-Delbrück distribution by the method of maximum likelihood was proposed long ago [8], [12], [31], [32]. Using well known explicit formulas, the method has been implemented [11], [14], [33]. In [15] it was shown that similar algorithms apply not only to the classical Luria-Delbrück distribution (in which division times are exponentially distributed), but also to the so-called Haldane model in which distribution times are supposed constant [34], [35]. The situation here is only slightly different. Instead of being considered as a sample of a fixed Luria-Delbrück distribution, mutant counts can be viewed as independent realizations of different distributions. Denote by 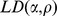 a Luria-Delbrück distribution with expected number of mutations 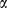 and relative fitness 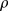. If a pair mutant count – final number 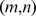 has been observed, 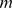 is viewed as a realization of the 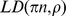, and the likelihood is computed accordingly. Thus the pair 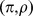 is jointly estimated, as the pair 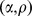 in the constant final number case.

Here is the mathematical model: for each experiment a pair of numbers giving the number of mutants and the final number of cells is obtained. An experiment is modelled by a couple 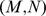 of random variables, where 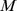 represents the number of mutants and 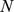 the total number of cells at the end of the experiment. The conditional distribution of 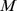 knowing 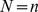 is assumed to be the generalized Luria-Delbrück distribution 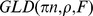. The notation is that of [15]: the expected number of mutations 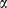 is the product of 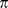 by the expected final number of cells, the relative fitness (ratio of the growth rate of the population of normal cells divided by that of mutants) is 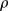, and the distribution of mutant division times is given by 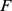. As in [15], we assume that a model has been chosen for the distribution of division times, so that only the mutation probability 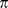 and the relative fitness 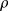 are to be estimated.

The sample size being 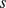, for 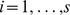 experiment number 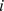 has yielded a couple 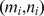, where 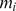 is the mutant count and 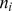 is the final number of cells. As in [14], [15], we denote by 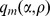 the probability of a mutant count equal to 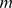, under the Luria-Delbrück distribution with parameters 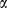 (expected number of mutations) and 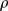 (relative fitness). The computation algorithms of the 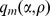 are well known and will not be reproduced here: see [12], [14], [15]. With that notation, the mutant count at the end of the 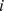-th experiment is equal to 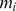 with probability 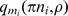. No assumption being made on the final counts, we consider the 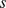-tuple of mutant counts 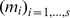 as a realization of a sample of independent random variables.

The log-likelihood is:
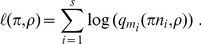
(4)


The computation of the gradient and Hessian of 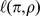 are only slightly different from those needed for the calculation of the maximum likelihood estimates of 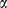 and 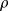 in the classical case [12], [14]. In the formulas below, be shall omit the dependence in 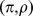 for clarity. The first and second derivatives of 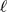 are evaluated at 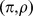, those of 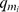 are evaluated at 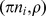. The gradient is computed by:
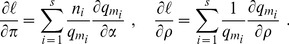
(5)


The Hessian is computed by:
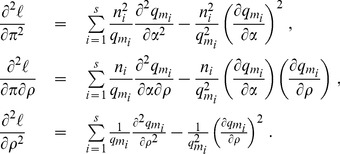
(6)


The first and second derivatives of 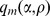 in 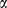 and 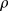 are obtained by recursive algorithms that will not be reproduced here [12], [14].

It is a well known fact in statistics, that the most easy looking maximum likelihood problem usually conceals algorithmic difficulties: numeric instability, bad conditionning of the Hessian, etc. [36]. Here, the procedure looks straightforward from (5) and (6): solving the gradient by a quasi-Newton or conjugate gradient method should be done quite efficiently at low computing cost. However, depending on the values in the sample, some optimization techniques may be more efficient than others. For the results described in this article, we have used the statistical software R [22], and compared several optimization algorithms: quasi-Newton, BFGS, conjugate gradient, simulated annealing [37]. The calculation of the Hessian at the maximum likelihood solution, which is needed to output asymptotic variances poses a numerical problem, already signalled in [14]. For the results of the article a numeric evaluation of the Hessian was used instead of (6) [37]. In File S1, only the simplest method has been included: it consists in solving the gradient by the Raphson-Newton method, from (5) and (6). It is not the best method by far. We are presently working on an optimized implementation, to be included in a forthcoming R package.

### Model for simulations

In the simulation study reported in the Results section, we have chosen to draw samples of final numbers according to a log-normal distribution with fixed expectation 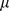 and coefficient of variation 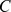. Other similarly shaped distributions could have been used: gamma, inverse Gaussian, Weibull, etc. Our choice of the log-normal was motivated by fitting real data, and by previously published results: see [16] and references therein.

If some value of the mutation rate 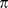 has been fixed, and the final number of cells 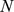 has been simulated, a mutant count can be drawn according to a Luria-Delbrück distribution with expected number of mutations 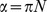 and relative fitness 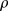. As explained in [15], an additional choice must be made: that of a probability distribution for division times. Neither of the two extreme choices that leads to computable versions of the Luria-Delbrück distribution (exponential and constant division times) is realistic. We have chosen the same distribution as in [15]: the best adjustment on Kelly and Rahn's observation on *Bacterium aerogenes* [38].

Simulations have been conducted for different sets of parameters. Results are reported for the following values, considered as representative:
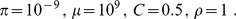



One thousand samples of size 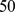 of pairs (mutant counts – final numbers) were simulated. For each sample, six estimates of 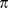 were computed, together with their theoretical standard deviation.

- *Classical methods*: the estimate of the expected number of mutations 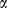 was computed by two different methods: the 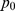-method [1], [5], [28], and the maximum likelihood (ML) method [12], [31], [32], both applied to the sample of mutant counts. Dividing by the expected final number 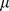, assumed to be known, leads to two estimates for 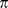.
- *unbiased estimates*: to each of the two previous estimates, the unbiasing formulas (1) and (3) were applied, assuming that the true value of the coefficient of variation was known which lead to two more estimates of 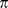. There again, the expected final number 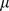 was supposed to be known, as well as the coefficient of variation 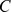.

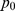
 *-method on the pairs*: no prior information being assumed, the maximum likelihood determination of 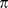 by the 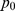-method was applied to the sample of pairs mutant counts – final numbers.
- *maximum likelihood for* 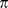 *and* 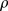: taking againg the sample of pairs with no prior information, a joint estimation for 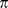 was obtained.

### Treatment for published datasets

We have reexamined data from David [17], and Werngren & Hoffner [21].

The data in Table 1 of [17] are not detailed, so only the 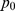-method could be applied. The bias correction (1) was applied, using a coefficient of variation of 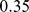 (estimated from Table 2 in the same reference).

Table 2 of [17] shows 10 pairs mutant counts – final numbers. All possible estimates were computed together with their confidence intervals. However, it must be remarked that standard deviation computations rely upon asymptotic results, and do not apply to such a small sample.

In Table 1 of [21] mutant counts are explicitly given. The maximum likelihood estimate with exponential division time was computed, then unbiased using a coefficient of variation of 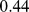 (estimated from the given final counts).

## Supporting Information

File S1 **File S1 is a script of the R functions that have been used for the simulation experiments described here.** It is a preliminary version of a forthcoming R package. The functions have not been protected nor optimized.
(R) Click here for additional data file.

## References

[1] Luria DE, Delbrück M (1943) Mutations of bacteria from virus sensitivity to virus resistance. Genetics 28: 491–511. 17247100 10.1093/genetics/28.6.491 PMC1209226

[2] Kendal WS, Frost P (1988) Pitfalls and practice of Luria-Delbrück fluctuation analysis: a review. Cancer Res 48: 1060–1065. 3277705

[3] Stewart FM, Gordon DM, Levin BR (1990) Fluctuation analysis: the probability distribution of the number of mutants under different conditions. Genetics 124: 175–185. 2307353 10.1093/genetics/124.1.175 PMC1203904

[4] Stewart FM (1994) Fluctuation tests: how reliable are the estimates of mutation rates? Genetics 137: 1139–1146. 7982567 10.1093/genetics/137.4.1139 PMC1206060

[5] Foster PL (2006) Methods for determining spontaneous mutation rates. Methods Enzymol 409: 195–213. 16793403 10.1016/S0076-6879(05)09012-9 PMC2041832

[6] Pope CF, O'Sullivan DM, McHugh TD, Gillespie SH (2008) A practical guide to measuring mutation rates in antibiotic resistance. Antimicrob Agents Chemother 52: 1209–1214. 18250188 10.1128/AAC.01152-07 PMC2292516

[7] Jin JL, Wei G, Yang WQ, Zhang HQ, Gao PJ (2012) Discussion on research methods of bacterial resistant mutation mechanisms under selective culture-uncertainty analysis of data from the Luria-Delbrück fluctuation experiment. Science China, Life sciences 55: 1007–1021. 23160830 10.1007/s11427-012-4395-7

[8] Sarkar S, Ma WT, v H Sandri G (1992) On fluctuation analysis: a new, simple and efficient method for computing the expected number of mutants. Genetica 85: 173–179. 1624139 10.1007/BF00120324

[9] Jones ME (1994) Luria-Delbrück fluctuation experiments; accounting simultaneously for plating efficiency and differential growth rate. J Theo Biol 166: 355–363. 10.1006/jtbi.1994.1032 8159019

[10] Jaeger G, Sarkar S (1995) On the distribution of bacterial mutants: the effects of differential fitness of mutants and non-mutants. Genetica 96: 217–223.

[11] Zheng Q (2002) Statistical and algorithmic methods for fluctuation analysis with SALVADOR as an implementation. Math Biosci 176: 237–252. 11916511 10.1016/s0025-5564(02)00087-1

[12] Zheng Q (2005) New algorithms for Luria-Delbrück fluctuation analysis. Math Biosci 196: 198–214. 15950991 10.1016/j.mbs.2005.03.011

[13] Gerrish PJ (2008) A simple formula for obtaining markedly improved mutation rates estimates. Genetics 180: 1773–1778. 18832356 10.1534/genetics.108.091777 PMC2581975

[14] Hamon A, Ycart B (2012) Statistics for the Luria-Delbrück distribution. Elect J Statist 6: 1251–1272.

[15] Ycart B (2013) Fluctuation analysis: can estimates be trusted? PLoS One 8: e80958. 24349026 10.1371/journal.pone.0080958 PMC3857183

[16] Koutsoumanis KP, Lianou A (2013) Stochasticity in colonial growth dynamics of individual bacterial cells. Appl Environ Microbiol 79: 2294–2301. 23354712 10.1128/AEM.03629-12 PMC3623257

[17] David HL (1970) Probability distribution of drug-resistant mutants in unselected populations of *Mycobacterium tuberculosis* . Appl Microbiol 20: 810–814. 4991927 10.1128/am.20.5.810-814.1970 PMC377053

[18] de la Iglesia F, Martínez F, Hillung J, Cuevas JM, Gerrish PJ, et al (2012) Luria-Delbrück estimation of turnip mosaic virus mutation rate in vivo. J Virol 86: 3386–3388. 22238294 10.1128/JVI.06909-11 PMC3302333

[19] Angerer WP (2001) An explicit representation of the Luria-Delbrück distribution. J Math Biol 42: 145–174. 11261316 10.1007/s002850000053

[20] Komarova NL, Wu L, Baldi P (2007) The fixed-size Luria-Delbrück model with a nonzero death rate. Math Biosci 210: 253–290. 17583754 10.1016/j.mbs.2007.04.007

[21] Werngren J, Hoffner SE (2003) Drug susceptible *Mycobacterium tuberculosis* Beijing genotype does not develop motation-conferred resistance to Rifampin at an elevated rate. J Clin Microbiol 41: 1520–1524. 12682139 10.1128/JCM.41.4.1520-1524.2003 PMC153924

[22] R Development Core Team (2008) R: A Language and Environment for Statistical Computing.R Foundation for Statistical Computing, Vienna, Austria. URL http://www.R-project.org. ISBN 3-900051-07-0.

[23] Lea DE, Coulson CA (1949) The distribution of the number of mutants in bacterial populations. J Genetics 49: 264–285. 24536673 10.1007/BF02986080

[24] Tan WY (1982) On distribution theories for the number of mutants in cell populations. SIAM J Appl Math 42: 719–730.

[25] Dewanji A, Luebeck EG, Moolgavkar SH (2005) A generalized Luria-Delbrück model. Math Biosci 197: 140–152. 16137718 10.1016/j.mbs.2005.07.003

[26] Ycart B (2014) Fluctuation analysis with cell deaths. J Appl Probab Statist 9: 12–28.

[27] Ford CB, Shah RR, Maeda MK, Gagneux S, Murray MB, et al (2013) *Mycobacterium tuberculosis* mutation rate estimates from different lineages predict substantial differences in the emergence of drug-resistant tuberculosis. Nature Genetics 45: 784–790. 23749189 10.1038/ng.2656 PMC3777616

[28] Fu J, Li IC, Chu EHY (1982) The parameters for quantitative analysis of mutation rates with cultured mammalian somatic cells. Mut Research 105: 363–370. 10.1016/0165-7992(82)90108-7 7144794

[29] Wasserman L (2004) All of statistics: a concise course in statistical inference. Springer, New York.

[30] Dyke P (2001) An introduction to Laplace transforms and Fourier series. Springer, London.

[31] Ma WT, v H Sandri G, Sarkar S (1992) Analysis of the Luria-Delbrück distribution using discrete convolution powers. J Appl Probab 29: 255–267.

[32] Jones ME, Wheldrake J, Rogers A (1993) Luria-Delbrück fluctuation analysis: estimating the Poisson parameter in a compound Poisson distribution. Comput Biol Med 23: 525–534. 8306630 10.1016/0010-4825(93)90099-m

[33] Hall BM, Ma C, Liang P, Singh KK (2009) Fluctuation Analysis CalculatOR (FALCOR): a web tool for the determination of mutation rate using Luria-Delbrück fluctuation analysis. Bioinformatics 25: 1564–1565. 19369502 10.1093/bioinformatics/btp253 PMC2687991

[34] Sarkar S (1991) Haldane's solution of the Luria-Delbrück distribution. Genetics 127: 257–261. 2004702 10.1093/genetics/127.2.257 PMC1204353

[35] Zheng Q (2007) On Haldane's formulation of the Luria-Delbrück mutation model. Math Biosci 209: 237–252. 10.1016/j.mbs.2007.03.003 17462675

[36] Gupta NK, Mehra RK (1974) Computational aspects of maximum likelihood: estimation and reduction in sensitivity function calculations. IEEE Trans Automatic Control 19: 774–783.

[37] Nocedal J, Wright S (2006) Numerical optimization. Springer, New-York, 2^nd^ edition.

[38] Kelly CD, Rahn O (1932) The growth rate of individual bacterial cells. J Bacteriol 23: 147–153. 16559540 10.1128/jb.23.2.147-153.1932 PMC533308

