# Correction: Unbiased Estimation of Mutation Rates under Fluctuating Final Counts

**DOI:** 10.1371/journal.pone.0173143

**Published:** 2017-03-13

**Authors:** Adrien Mazoyer, Bernard Ycart, Nicolas Veziris

The authors would like to update their published article with the following information in response to the online comment posted by jwerngren on 28 Jun 2016:

## The *Mycobacterium tuberculosis* data from [1]

In the results section of [2], the following is said:

Table 2 reports mutation rate estimates by the ML method, from data in Table 1 of Werngren & Hoffner [1]. The second column contains the authors’ estimates, calculated by Luria & Delbrück method of the mean. The next two columns contain the unbiased ML estimate and its 95% confidence interval. Except for two strains, the authors’ estimate is outside the confidence interval. Here, the method of the mean used by the authors has underestimated the mutation rate, because of the very small number of jackpots in the data. The main conclusion of [1] was that no significant difference had been observed between non-Beijing strains (first seven lines) and Beijing strains (last six lines). Actually, the average mutation rate over the first seven lines is 4.37 × 10^−8^, over the last six lines it is 2.69 × 10^−8^. The difference is significant at threshold 5% (Welsh Two Sample t-test, P = 0.047).

Since [2], some progress has been made in the statistical methods of estimation for mutation rates. State of-the-art algorithms have been included in the new “flan” R package that had been announced in the conclusion of [2]. It is now available on the CRAN web site [3]. The data from [1] have been included in that package, as variable werhoff. That they correspond to the columns of Table 1 in [1], in the same order, can be checked by the R commands:

library("flan")

wh <- werhoff[["samples"]]

lapply(wh,function(w){table(w$mc)})

In particular, the assertion that the first seven lines of Table 2 in [2] correspond to non-Beijing strains in Table 1 of [1] is correct.

Using the flan package, anyone can easily check the authors’ results. Here are the R commands:

Wcvfn <- werhoff$cvfn

piestim <- unlist(lapply(wh,function(w){mutestim(w$mc,mfn = w$mfn,cvfn = Wcvfn)$mutprob}))

nonbeijing <- piestim[1:7]

beijing <- piestim[8:13]

t.test(nonbeijing,beijing,alternative = "greater")

Due to the fact that the algorithms are different from those used in [2], the results are slightly different. The mean mutation rate over non-Beijing strains is now found to be 4.02 × 10^−8^ instead of 4.37 × 10^−8^, over Beijing strains it is 2.37 × 10^−8^ instead of 2.69 × 10^−8^. The p-value of the Welsh two-sample t-test is 0.034 instead of 0.047. However, the conclusion remains unchanged: *the mean mutation rate over the seven non-Beijing strains is significantly greater at threshold 0*.*05 than the mean mutation rate over the six Beijing strains*.

The second passage in which [1] is referred to in [2] is in the discussion section:

The demonstration is even more striking in Werngren and Hoffner’s paper. They compared mutation rate between Beijing and non Beijing *M*. *tuberculosis* strains and concluded that it was not different and thus could not explain the strong association between Beijing strains and multidrug resistance phenotype. However we re-calcutated the mutation rate and showed that it was significantly higher for Beijing vs. non-Beijing strains. This result is consistent with a recent paper [4] showing that lineage 2 (Beijing) *M*. *tuberculosis* strains have a higher mutation rate than lineage 4 (non-Beijing) strains.

There is indeed an error here, for which the authors must apologize: “Beijing” and “non-Beijing” have been inadvertently swapped in the discussion, thus contradicting the (correct) assertion of the results section.

Thus the discussion section should be modified. The present results are in fact in opposition with those of [4] since they show that the average mutation rate of the selected non-Beijing isolates was higher than that of the Beijing isolates. One possible explanation could be that these differences do not reflect a general property of Beijing strains but rather an individual property of the strains that have been selected for each experiment.

## The precision of mutation rate estimates

In his comment, J. Werngren argues that:

In our 2003 study, we concluded that the reproducibility of the fluctuation assay, if performed under strictly standardized conditions, seem to be within the power of ten.

Later, about the discrepancies detected in [2] J. Werngren observes that:

Noteworthy, their mutation rates still differ less than the power of ten between the two groups of strains but also when compared to the 2003 calculations.

These two assertions, together with the conclusion of the comment, seem to imply that a ten-fold difference between two mutation rates should not be considered as significant. This is not acceptable from a statistical point of view. It is precisely the role of statistics to reduce uncertainty, and ground decisions to be taken on data sets on rigorous bases.

In Table 1 of [5], an extensive list of “Published rates for evolution of drug resistance to various antibiotics in *M*. *tuberculosis* and related mycobacteria” is given. Indeed, it shows huge discrepancies: the mutation rates given in that list vary from 10^−5^ to 10^−11^. Does this mean that these huge differences correspond to the reality of true mutation rates? The authors do not believe so.

Even though it might seem a disturbing fact for the experimentalist, there is no way to actually *measure* a mutation rate. Understood as the proportion of cell divisions with mutant offspring, it can only be *estimated* using a mathematical mutation model, and a statistical estimation method. As stated at the beginning of the discussion section in [2]:

In any estimation problem, three levels must be distinguished: the reality which is and will remain unknown, the mathematical model which involves more or less realistic hypotheses, and the estimation method. Minimal requirements for an estimator are consistence (outputs should be close to the unknown value of the parameter), and a computable asymptotic variance (to allow statistical inference). Since there is no way to validate all mathematical hypotheses that define the model, another quality is desirable: robustness. Indeed, designing an estimator for a given model and applying it to a different one usually induces a bias: the smaller the bias, the more robust the estimator.

Some of the non-realistic hypotheses of the classical mathematical models used in fluctuation analysis are listed in [6], p. 1211: cells do not die, mutants and normal cells have the same growth rate, etc. More have been considered since. The mutestim function of flan takes into account cell deaths as well as differential growth rates, final numbers, and division time distributions. More complete models are currently under study, and new functionalities will be included in the future versions of flan.

Regarding estimation methods, only three of them meet both requirements of consistency and computable asymptotic variance: the original P0-method of Luria and Delbrück [7], the GF method of [8–10], and of course the Maximum Likelihood method, initially proposed for the Luria-Delbrück model by Sarkar, Ma, and Sandri [11, 12]. All three are implemented in flan. Other methods have been proposed: see [6,13]. They should not be used. In particular, the Luria- Delbrück method of the mean used in [1] and many other papers is not consistent, and very sensitive to the size of jackpots. Monte Carlo simulations show that for a given mutation rate, its estimates over random samples can be off-target by several orders of magnitude.

For a given data set, using any of the three valid estimation methods, and taking into account or not cell deaths, differential growth rates, final numbers, and division time distributions, different mutation rates estimates will be obtained. Admittedly, mutation rate estimates on the same data set using different methods and modeling assumptions, usually differ by less than 50%. This is far from the ten-fold difference mentioned by J. Werngren. However, the authors still believe that the importance of the public health issue justifies computing as precise and realistic estimates as possible.

## Conclusion

There was indeed an error in the discussion section of [2]. The authors renew their apologies, and thank J. Werngren for his vigilance. The authors of [1] must also be thanked for publishing a very useful and complete data set.

However, the authors maintain that the results of [2], and in particular their analysis of the data in [1] remain valid. The statistical methods described in [2] have subsequently been implemented in the R package flan [3]. Researchers who need fluctuation analysis, in particular in the field of drug resistance, are welcome to use it. The authors believe that this could increase the precision of mutation rate estimates, and give more firm grounds to statistical decisions.
